# Prognostic significance and immunoinfiltration analysis of genes associated with epithelial-mesenchymal transition and energy metabolism in bladder urothelial carcinoma

**DOI:** 10.18632/aging.205242

**Published:** 2023-11-24

**Authors:** Yifan Qiu, Wei Ye, Chao Wang, Jin Zang

**Affiliations:** 1 The First Affiliated Hospital of Soochow University, Suzhou 215006, Jiangsu, China

**Keywords:** EMT, energy metabolism, CSPG4, prognosis, immune microenvironment

## Abstract

Background: Epithelial-mesenchymal transition (EMT) and aberrant energy metabolism are pivotal biological processes in tumor progression, significantly impacting tumor prognosis. However, the relationship between EMT, energy metabolism, and the immune microenvironment in bladder urothelial carcinoma (BLCA) remains inadequately understood.

Methods: Bladder cancer samples from The Cancer Genome Atlas were categorized into two groups via clustering analysis to elucidate disparities in expression, prognostic significance, and immune infiltration of genes associated with EMT and energy metabolism between these groups. Key genes associated with EMT and energy metabolism in BLCA were identified through Cox multifactorial regression analysis, immune infiltration analysis, etc. Subsequently, their prognostic significance in BLCA was validated.

Results: Cluster analysis revealed significant differences in the expression of genes associated with EMT and energy metabolism between the two groups. Group 2 exhibited significantly improved overall survival and progression-free survival compared to Group 1. Chondroitin sulfate proteoglycan 4 (CSPG4) emerged as the most critical gene associated with EMT, energy metabolism, prognosis, and immune infiltration in BLCA. Immunohistochemical assays demonstrated differential expression of CSPG4 in bladder tumors and normal bladder tissues, with high CSPG4 expression correlating with a poorer BLCA prognosis. Furthermore, CSPG4 exhibited an association with the immune checkpoint molecule programmed death-1 (PD1) in BLCA.

Conclusions: EMT and energy metabolism exert pivotal influences on the immune microenvironment in BLCA. CSPG4 holds promise as a prognostic biomarker for patients with BLCA, offering valuable insights into potential immunotherapeutic strategies for this patient population.

## INTRODUCTION

Bladder urothelial carcinoma (BLCA) is a prevalent urogenital malignancy characterized by significant morbidity and mortality. With approximately 573,278 new cases of BLCA and 212,536 associated deaths reported worldwide, it has emerged as a substantial global health concern [1]. BLCA can be categorized into two primary clinical subtypes: muscle-invasive bladder cancer and non-muscle-invasive bladder cancer. The latter, non-muscle-invasive bladder cancer, constitutes the most prevalent clinical subtype, distinguished by a notable recurrence rate but a relatively low mortality rate [2]. The standard therapeutic approach for non-muscle-invasive bladder cancer typically involves transurethral resection of the bladder tumor, often complemented by postoperative bladder perfusion chemotherapy. In contrast, the management of muscle-invasive bladder cancer necessitates more aggressive interventions, such as radical resection or cisplatin-based neoadjuvant chemotherapy [3]. Although surgical treatment and chemotherapy have shown promising results in the initial management of patients with BLCA, the likelihood of tumor recurrence remains high, exceeding 50 percent after five years of treatment. Additionally, there is a notable risk of tumor progression [4]. Therefore, exploring safe and efficacious treatment options to enhance the prognosis of patients with BLCA holds significant importance.

In recent years, immunotherapy has exhibited promising outcomes when employed for BLCA treatment, particularly with the initial use of Bacillus Calmette–Guérin. Its primary mechanism of action involves the activation of CD4+ T cells in the tumor microenvironment to stimulate the adaptive immune response of T helper 1 cells [5]. Recent studies have highlighted the efficacy of immune checkpoint inhibitors (ICIs) in advanced BLCA. It has been established that programmed death-1 (PD1), cytotoxic T lymphocyte-associated antigen-4, and programmed cell death-ligand 1 (PD-L1) serve as crucial targets for immunotherapeutic agents. Notably, patients with BLCA exhibiting high PD-L1 expression demonstrate a 6.2% higher five-year overall survival (OS) rate with immunotherapy compared to chemotherapy. Therefore, further exploration of the clinical application of BLCA immunotherapy is warranted [6, 7]. The identification of relevant immune-related prognostic markers through screening will offer additional avenues for enhancing BLCA immunotherapy [8].

Epithelial-mesenchymal transition (EMT) is a biological process wherein epithelial cells undergo a transformation into cells displaying a mesenchymal phenotype through specific steps. This process entails cytoskeletal remodeling, resulting in mitochondrial division, which generates a substantial energy supply. This energy, in turn, fuels EMT—a mechanism of paramount significance in tumor progression [9]. Disrupted energy metabolism, encompassing perturbations in glycolysis and the tricarboxylic acid cycle, can bolster tumor proliferation and migration [10]. A growing body of evidence underscores the intimate connection between EMT and energy metabolism, as elucidated in studies concerning breast, thyroid, and lung cancers. Through aerobic glycolysis (the Warburg effect), tumor cells modulate glucose metabolism to generate the requisite energy for facilitating EMT. Concurrently, EMT fosters the synthesis of cholesterol and fatty acids in tumor cells, amplifying their metabolic processes and thereby propelling tumor progression [11]. In colorectal cancer and gastric cancer, the co-occurrence of aberrant energy metabolism and EMT progression correlates with elevated mortality rates and unfavorable prognoses [12–14]. A recent study demonstrated that EMT is associated with immune evasion in tumors and can modulate the expression of PD-L1 through molecules such as nuclear factor-kappa B (NF-κβ) and transforming growth factor-β (TGF-β), exhibiting a bidirectional relationship [15]. Furthermore, targeting the EMT pathway and employing EMT-related gene therapy have shown promise in interfering with tumor cell metabolism and enhancing the prognosis of patients with breast cancer [16]. Moreover, the aberrant energy metabolism of tumor cells can lead to alterations in the tumor immune microenvironment (TIME), contributing to immune evasion [17]. Similarly, investigations have revealed that EMT in BLCA can promote tumor progression and influence the prognosis of patients with BLCA by modulating immune cell infiltration in the TIME [18]. Aberrant energy metabolism also plays a pivotal role in BLCA progression, primarily through the regulation of tumor cell respiration, glycolysis, and mitochondrial metabolism to meet the energy demands of tumor proliferation and migration. Inhibition of glycolysis has demonstrated the potential to downregulate BLCA progression [19]. In a previous study, we discovered a mutual regulatory relationship between EMT and energy metabolism in BLCA [20]. Therefore, identifying genes associated with EMT and energy metabolism and examining their respective roles within the TIME holds substantial significance for the treatment of malignant tumors [21]. However, a comprehensive understanding of the effects of EMT and energy metabolism-related genes on the prognosis and TIME in patients with BLCA remains lacking.

Chondroitin sulfate proteoglycan 4 (CSPG4) has been demonstrated to exhibit aberrant expression in various tumor types, including soft tissue sarcoma, squamous cell carcinoma of the head and neck, breast cancer, and anaplastic thyroid cancer. Furthermore, CSPG4 has been shown to play a pivotal role in promoting tumor proliferation and metastasis [22–24]. Simultaneously, CSPG4 serves as a promising therapeutic target for cancer. Targeting CSPG4 disrupts the TIME, thus impeding the progression and metastasis of triple-negative breast cancer [25]. CSPG4 also exerts a significant influence on the prognosis of patients with tumors; its high expression levels are associated with unfavorable outcomes in patients with glioblastoma multiforme. Consequently, CSPG4 holds promise as a prognostic marker in the context of cancer [26]. However, the expression of CSPG4 in BLCA and its role as a prognostic biomarker necessitate further investigation.

In this study, we comprehensively explored the expression profiles of genes associated with EMT and energy metabolism in BLCA, examining their associations with prognosis and the TIME. Additionally, we conducted a cluster analysis of bladder cancer samples from the Cancer Genome Atlas (TCGA), categorizing them into two groups. We then explored the correlations between these groups and genes associated with EMT and energy metabolism, aiming to analyze the differential expression and roles of EMT and energy metabolism-related genes in various BLCA samples. Ultimately, through methods such as Cox multi-factor regression analysis, we identified CSPG4 as the most representative factor associated with both EMT and energy metabolism in BLCA. High expression of CSPG4 in BLCA was determined to be associated with poor prognosis in patients with BLCA and was closely linked to PD1 expression in BLCA.

## MATERIALS AND METHODS

### Samples and datasets

Clinical information pertaining to patients with BLCA was sourced from TCGA database via the Genomic Data Sharing pathway (https://portal.gdc.cancer.gov/). Expression data pertaining to CSPG4 in BLCA was extracted from the GSE3167 dataset in the Gene Expression Omnibus (GEO) database (https://www.ncbi.nlm.nih.gov/geo/). To further elucidate the expression levels and prognostic implications of CSPG4, we conducted validation through a bladder cancer tissue microarray. This microarray consisted of 68 BLCA tissue samples (HBlaU108Su01) and 40 corresponding normal bladder tissues, acquired from Shanghai Outdo Biotech Co. After excluding data pertaining to patients with lost follow-up visits and those undergoing tissue dewaxing, we analyzed a total of 55 bladder cancer tissues and 31 adjacent non-tumor tissues. Ethical review and approval for this study were obtained from the Ethics Committee of Shanghai Outdo Biotech Co.

### Clustering analysis

The “ConsensusClusterPlus” R package (version 1.54.0) was employed to analyze the expression levels of selected regulators associated with EMT and energy metabolism in patients with BLCA. A maximum of six groups were defined, and this process was repeated 100 times, each time utilizing 80% of the total sample. Subsequently, the principal components were examined using the “ggplot2” R package for visualization.

### Gene set enrichment analysis (GSEA)

The “ClusterProfiler” R package was employed for GSEA [27].

### Gene immune infiltration analysis

We employed the TIMER and CIBERSORT tools from the “immuneeconv” R package to validate our immune score evaluations. Additionally, we utilized the R Foundation for Statistical Computing (version 4.0.3, 2020) in conjunction with the ggplot2 and “pheatmap” R packages to present our analysis results.

### Prognostic analysis of CSPG4 in BLCA

We employed the Kaplan–Meier plotter and the PrognoScan database for external validation of the correlation between CSPG4 expression and survival. To predict future survival, we integrated both univariate and multivariate Cox regression methods. Additionally, we generated a nomogram to enhance the clarity of our analytical findings.

### Immunohistochemistry

Anti-CSPG4 antibody (bs-23788R) was purchased from Beijing Boosen Biotechnology Co., Beijing, China. Following the dewaxing and hydration of the tissue slices, antigen retrieval was performed, and the slices were allowed to cool to room temperature. Subsequently, they were incubated with a Bovine Serum Albumin (BSA) blocking solution for 30 min, followed by the addition of the monoclonal antibody at a 1:60 ratio and incubation overnight at 4° C. Afterward, the slices were rewarmed at 37° C for 30 min and then subjected to three washes with Phosphate Buffer Solution (PBS) (pH 7.2–7.6). Polyclonal Horseradish Peroxidase (HRP) anti-rabbit IgG was introduced and incubated at 37° C for 30 min, followed by another three PBS washes. Finally, 3,3’-Diaminobenzidine (DAB) was added to induce color development, and the specimens were observed under a microscope. The assessment of staining quality was based on both staining percentage and intensity. A score of four was assigned when the staining covered over 75% of the area, three for 50% to 75%, two for 25% to 50%, and one for 0% to 25%. Staining intensity was graded as three for high, two for medium, one for low, and zero for no staining. The overall staining score was obtained by multiplying the staining percentage and intensity scores.

### Statistical analyses

The Chi-square test was employed to evaluate the association between high and low CSPG4 expression in tumor and paraneoplastic tissues and clinicopathological features. Survival curves were generated through Kaplan–Meier analysis and assessed using the log-rank test. Statistical analyses were conducted using the IBM SPSS Statistics 25 software. Statistical significance was established at a threshold of P < 0.05, denoted as *P < 0.05, **P < 0.01 and ***P < 0.001.

### Data availability

The datasets presented in this study can be found in online repositories. The names of the repository/repositories and accession number(s) can be found in the article.

## RESULTS

### Expression of EMT- and energy metabolism-related genes in BLCA and normal tissues

**Figure 1:**
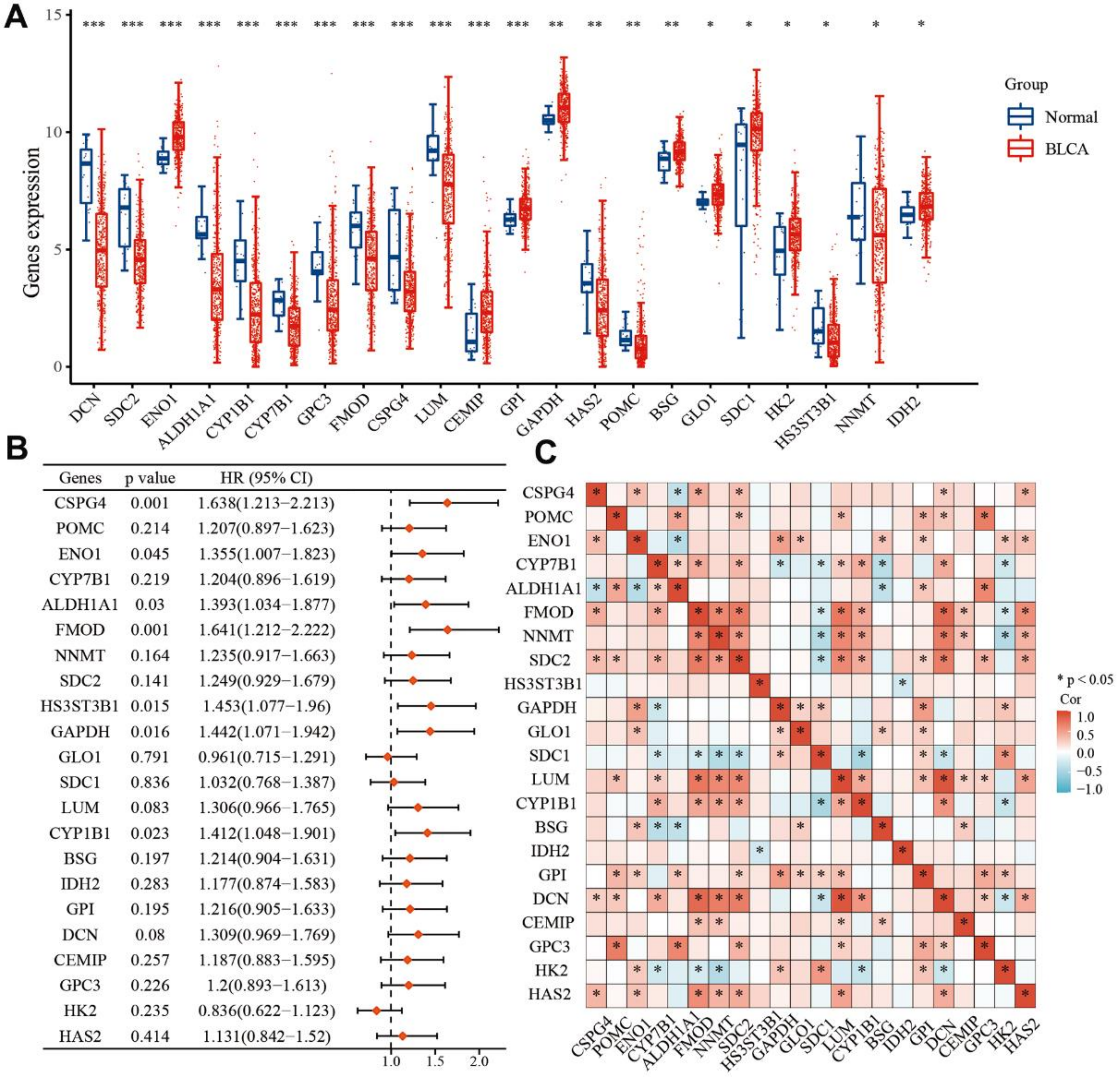
**Differential expression of 22 key EMT- and energy metabolism-related genes in BLCA.** (**A**) Expression of 22 EMT- and energy metabolism-related genes in BLCA tissue and normal bladder tissue. (**B**) OS forest map of 22 key EMT- and energy metabolism-related genes. (**C**) Correlation among the 22 key EMT- and energy metabolism-related genes.

EMT-related genes were sourced from the Molecular Signatures database [28], while energy metabolism-related genes were acquired from the Molecular Signatures database as well [29]. Initially, we screened a selection of 590 genes associated with energy metabolism in TCGA BLCA dataset, followed by a screening of 1,263 genes associated with EMT. Ultimately, we identified 22 key genes that were associated with both energy metabolism and EMT in BLCA, utilizing a combination of differential expression analysis and univariate analysis. Our analysis revealed that among these 22 genes, *ENO1*, *CEMIP*, *GPI*, *GAPDH*, *BSG*, *GLO1*, *SDC1*, *HK2*, and *IDH2* exhibited higher expression levels in BLCA compared to normal tissues. Conversely, *DCN*, *SDC2*, *ALDH1A1*, *CYP7B1*, *CYP1B1*, *GPC3*, *FMOD*, *CSPG4*, *LUM*, *HAS2*, *POMC*, *HS3ST3B1*, and *NNMT* displayed lower expression in BLCA compared to normal tissues (Figure 1A). Furthermore, we observed that low expression of *GLO1* and *HK2* correlated with a prognosis in patients with BLCA, and high expression of the remaining 20 genes was associated with an unfavorable prognosis (Figure 1B). Among the 22 genes, *SDC1*, *HK2*, and *CYP7B1* displayed negative associations with a greater number of genes, while the other 19 genes exhibited positive associations with a greater number of genes (Figure 1C).

### Consensus clustering analysis of EMT- and energy metabolism-related genes in BLCA

**Figure 2:**
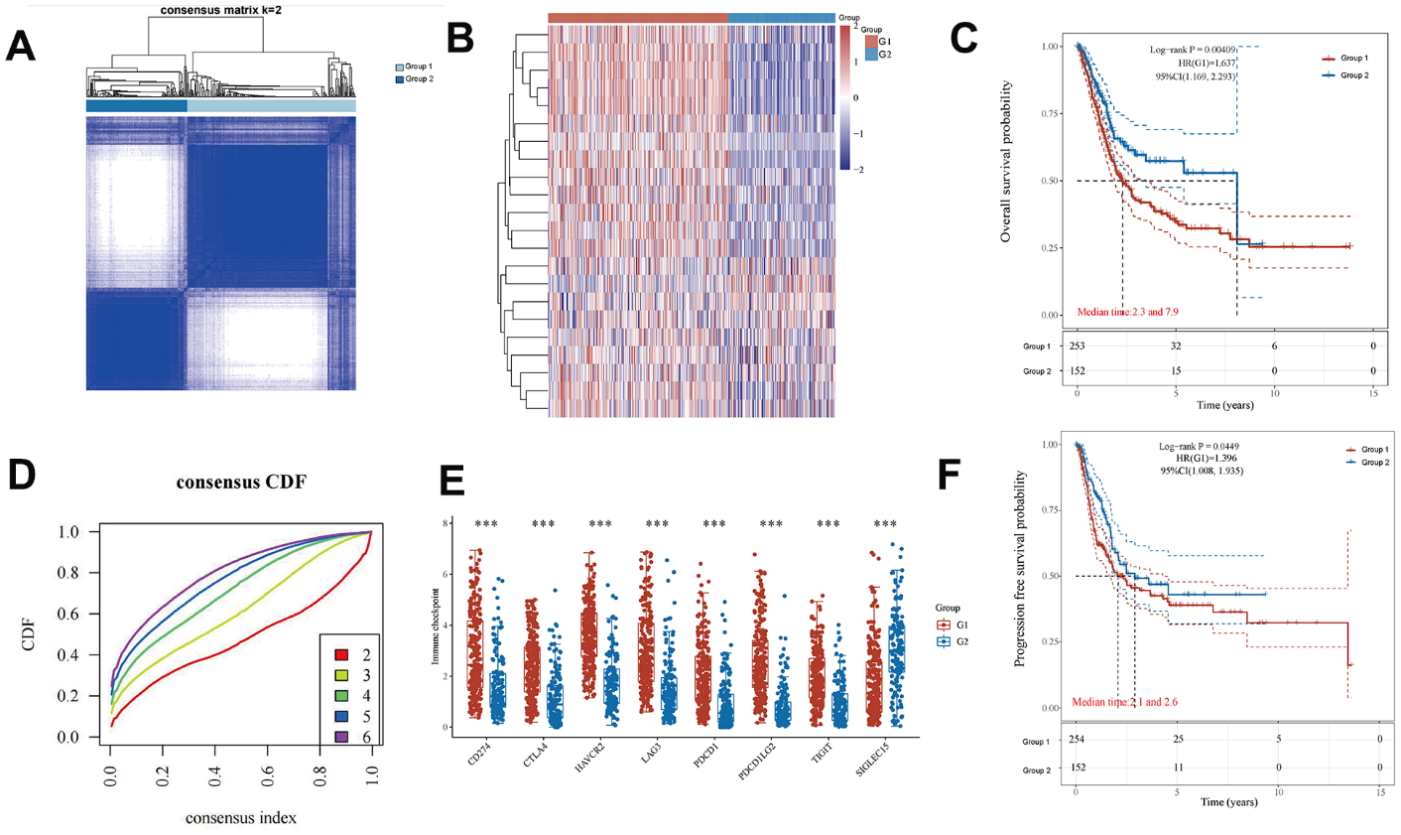
**Differential expression of EMT- and energy metabolism-related genes in two groups of BLCA samples.** (**A**) BLCA samples were divided into two groups using the consensus clustering method. (**B**) A heatmap of the differential expression of EMT- and energy metabolism-related genes. (**D**) Consensus distribution for each k value in the empirical CDF graph. (**E**) Differential expression of immune checkpoint-related genes between the two groups. (**C**, **F**) OS and progression-free survival in both groups.

Consensus clustering of EMT- and energy metabolism-related genes in BLCA primarily aided our investigation into the associations between EMT, energy metabolism, clinical characteristics, and the prognosis in patients with BLCA. This method facilitated the grouping of samples with higher similarity (indicated in blue) together, thereby distinguishing them from samples with lower similarity (depicted in white). Such clustering aids in gaining a deeper understanding of the dataset's inherent structure [30]. In Figure 2A, a clear partition is evident when k = 2. Moreover, the empirical cumulative distribution function (CDF) graph provides insights into the consensus distribution for various k values. Notably, when k = 2, the CDF curve exhibits the flattest profile, indicating a reduced likelihood of an inconsistent distribution [31]. Therefore, the model identifies the optimal number of groups, which is found to be k = 2. Consequently, the patients were categorized into two stable groups (Figure 2D), comprising 254 patient samples in Group 1 and 152 patient samples in Group 2. A heatmap revealed substantial differences in the expression of genes associated with EMT and energy metabolism between these two groups (Figure 2B). Furthermore, disparities in the expression of immune checkpoint-related genes between the groups were observed (Figure 2E). Finally, a comparison of OS and progression-free survival for patients in both groups revealed markedly improved outcomes for patients in Group 2 (Figure 2C, 2F). These results underscore the marked distinctions between these two sets of patient samples, further underscoring the differential expression of EMT- and energy metabolism-related genes in the two groups of BLCA samples.

### Correlation of EMT- and energy metabolism-related genes with BLCA immune cell infiltration

**Figure 3:**
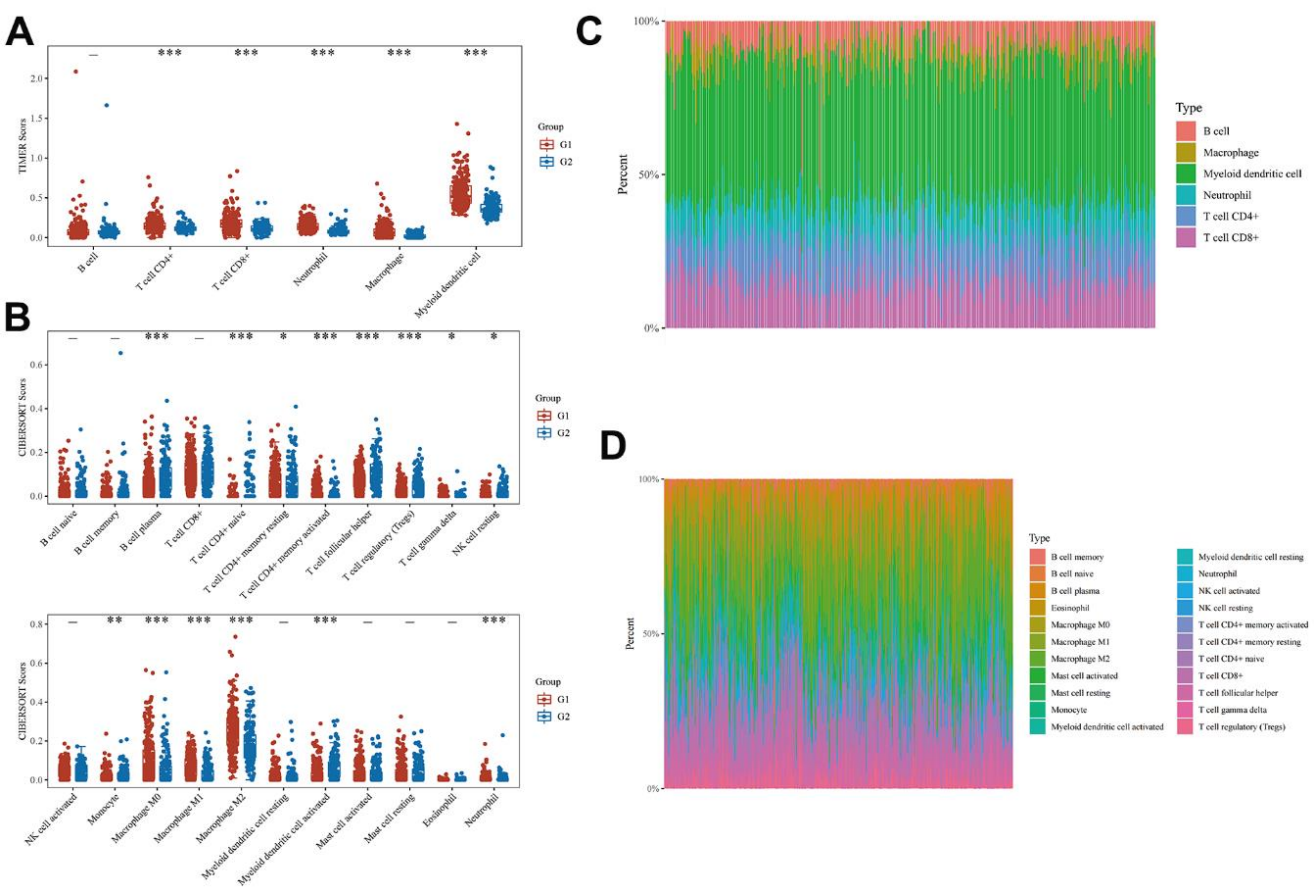
**Correlation between EMT- and energy metabolism-related genes and immune cell infiltration in BLCA.** (**A**) Differences in the expression of immune cells in the two groups were analyzed using the TIMER algorithm. (**B**) A heatmap of the percentages of immunoinfiltrating cells in both groups. (**C**) CIBERSORT algorithm was used to further analyze the differences in the expression of immune cells in the two groups. (**D**) A heatmap of the percentages of immunoinfiltrating cells in the two groups was further analyzed.

We categorized the BLCA samples into two groups, designated as Group 1 and Group 2, via cluster analysis. Subsequently, we assessed the correlation between these two groups and immune cells using the TIMER and CIBERSORT algorithms, respectively. Employing the TIMER algorithm, we observed differential expression of immune cells between the two groups. Specifically, the expression levels of CD4+ T cells, CD8+ T cells, neutrophils, macrophages, and myeloid dendritic cells in Group 1 surpassed those in Group 2 (Figure 3A). Figure 3C displays a heatmap showing the percentages of tumor-infiltrating immune cells in each group. We further employed the CIBERSORT algorithm to evaluate the differential expression of genes and immune cells between the two groups. Figure 3B, 3D illustrate the results in the form of heatmaps depicting the expression as percentages. These results substantiate the correlation between EMT- and energy metabolism-related genes in BLCA and tumor-infiltrating immune cells. Therefore, these genes exert a significant influence on the immune microenvironment in BLCA.

### Key prognostic biomarkers associated with immune infiltration of EMT- and energy metabolism-related genes in BLCA

**Figure 4:**
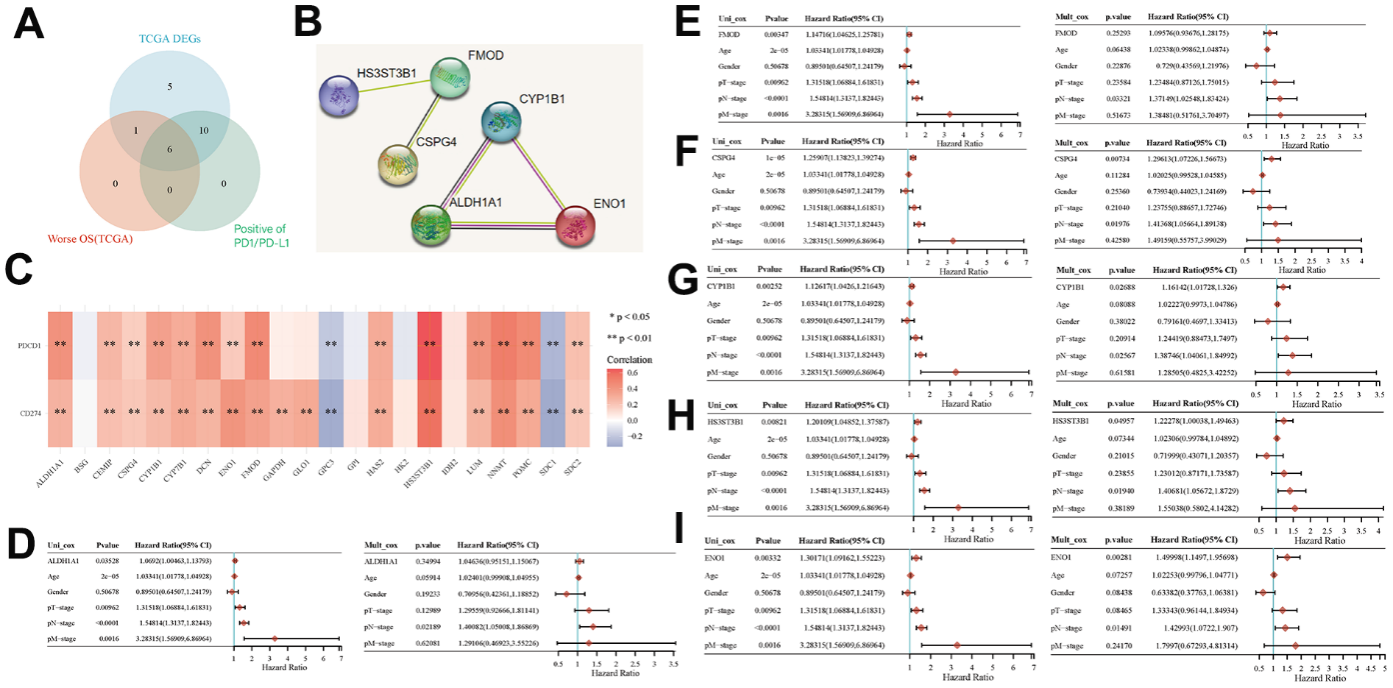
**Key prognostic biomarkers associated with immune infiltration of EMT- and energy metabolism-related genes in BLCA.** (**A**) Screening for genes associated with EMT and energy metabolism in BLCA that were positively correlated with PD1/PD-L1 expression by Venn mapping. (**B**) Interaction relationship of six genes was analyzed using the STRING database. (**C**) Correlation between the expression of EMT- and energy metabolism-related genes and PD1/PD-L1 expression. (**D**–**I**) Correlation between the expression of six genes and prognosis was analyzed via univariate and multivariate Cox analyses.

We screened TCGA database to identify six key genes that exhibited prognostic relevance in BLCA while being associated with EMT and energy metabolism. These six genes were *HS3ST3B1*, *FMOD*, *CSPG4*, *ALDH1A1*, *CYP1B1*, and *ENO1*, all of which displayed positive correlations with PD1/PD-L1 expression (Figure 4A). Furthermore, we employed the STRING database to investigate interactions involving these six genes. The results showed that *ALDH1A1*, *CYP1B1*, and *ENO1* genes were related, while *FMOD* affected HS3ST3B1 and CSPG4 (Figure 4B). Subsequently, we explored the association between PD1/PD-L1 expression and genes associated with EMT and energy metabolism. The results revealed a positive correlation between PD1/PD-L1 expression and most EMT- and energy metabolism-related genes, as evident in the heatmap, but a negative correlation with GPC3 and SDC1 expression (Figure 4C). Finally, we assessed the impact of HS3ST3B1, FMOD, CSPG4, ALDH1A1, CYP1B1, and ENO1 expression on the OS of patients with BLCA via univariate and multifactorial Cox regression analyses. Univariate COX analysis revealed that ALDH1A1 expression (p = 0.03528), FMOD expression (p = 0.00347), CSPG4 expression (p = 1e-05), CYP1B1 expression (p = 0.00252), HS3ST3B1 expression (p = 0.00821), and ENO1 expression (p = 0.00332) were all correlated with OS in patients. However, multifactorial Cox analysis indicated that only CSPG4 expression (p = 0.00734), CYP1B1 expression (p = 0.02688), HS3ST3B1 expression (p = 0.04957), and ENO1 expression (p = 0.00281) were associated with OS in patients (Figure 4D–4I). Given the high expression of PD1/PD-L1 in BLCA and its significance as a prognostic biomarker, it serves as a crucial target in BLCA immunotherapy [32, 33]. Therefore, the study of key genes linked to PD1/PD-L1 assumes substantial importance in the context of BLCA immunotherapy and patient prognosis.

### Clinical significance of key prognostic genes associated with EMT and energy metabolism in BLCA

**Figure 5:**
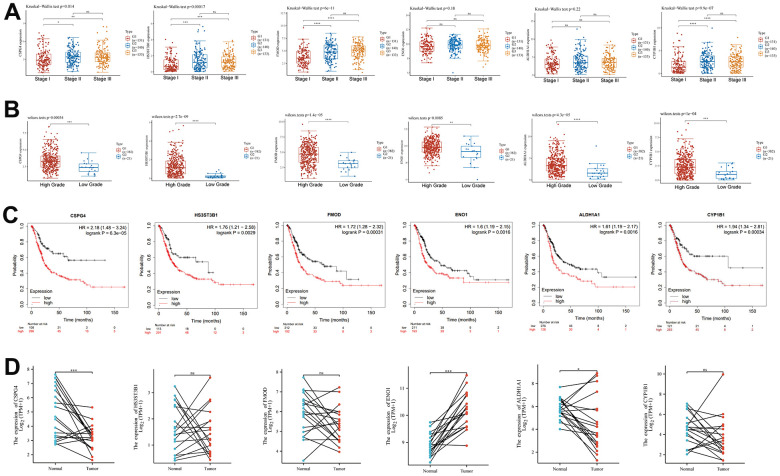
**Clinical significance of EMT- and energy metabolism-related key prognostic genes in BLCA.** (**A**) Expression of six key EMT- and energy metabolism-related genes in BLCA at different pathological stages. (**B**) Six key genes associated with EMT and energy metabolism were differentially expressed across the distinct BLCA grades. (**C**) Prognostic significance of the six key genes in BLCA was analyzed via Kaplan–Meier analysis. (**D**) Paired-sample analysis of the expression of the six key genes in BLCA tissues and corresponding paired normal tissues.

We analyzed the differential expression of six key genes associated with EMT and energy metabolism across distinct pathological stages of BLCA. The BLCA samples were categorized into three stages, comprising 131 cases in Stage I, 140 cases in Stage II, and 133 cases in Stage III. The results revealed significant differences in the expression of CSPG4 (p = 0.014), HS3ST3B1 (p = 0.00017), and FMOD (p = 6e-11) among the different BLCA pathological stages (Figure 5A). Subsequently, we examined the variations in the expression of these six key genes across different grades of BLCA, classifying the BLCA samples into two groups: high-grade BLCA and low-grade BLCA, with 382 cases in the former and 21 cases in the latter. The results demonstrated that HS3ST3B1, FMOD, CSPG4, ALDH1A1, CYP1B1, and ENO1 exhibited significantly higher expression levels in the high-grade BLCA group (Figure 5B). We further employed Kaplan–Meier analysis to assess the prognostic significance of these six key genes in BLCA samples, revealing that all six genes held statistical significance in predicting BLCA prognosis (p < 0.05) (Figure 5C). Lastly, paired sample analysis was conducted to investigate differences in the expression of the six key genes between tumors and their corresponding normal tissues. Among these genes, CSPG4, ENO1, and ALDH1A1 displayed statistically significant differences (Figure 5D). In summary, our comprehensive analysis highlighted that among the examined genes, only CSPG4 exhibited statistical significance across various aspects of BLCA, including immune infiltration, prognosis, pathological staging, grading, and paired sample analysis. Therefore, CSPG4 emerges as the most significant gene associated with EMT and energy metabolism, closely associated with immune infiltration and prognosis in BLCA.

### High CSPG4 expression is a prognostic biomarker for BLCA

**Figure 6:**
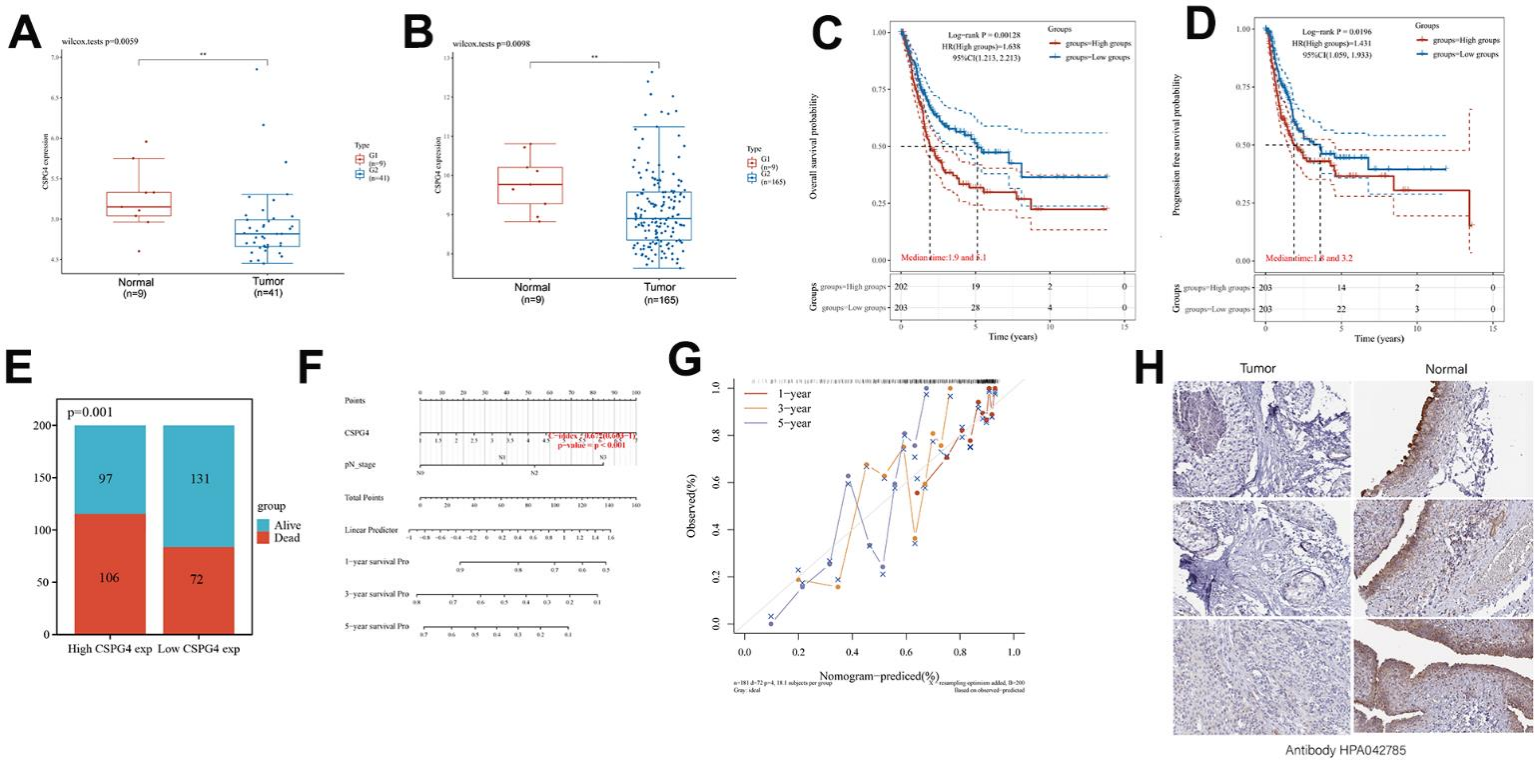
**High CSPG4 expression is a BLCA prognostic biomarker.** (**A**, **B**) CSPG4 expression in BLCA and normal tissues in the GSE3167 and GSE13507 datasets. (**C**, **D**) Association of CSPG4 expression with OS and progression-free survival in patients with BLCA. (**E**) Comparison of death and survival rates between high- and low-CSPG4 expression groups. (**F**, **G**) Plotting of nomogram and OS nomogram models based on the results of multivariate Cox regression analysis of CSPG4 expression. (**H**) Analysis of CSPG4 protein expression in BLCA and normal tissues using the Human Protein Atlas database.

The results of our prior analysis illustrated that CSPG4 exhibits differential expression in both BLCA and normal tissues, as well as when compared to paired normal tissues. To further substantiate this differential expression of CSPG4 between BLCA and normal tissues, we conducted analyses utilizing the GSE3167 and GSE13507 datasets from the GEO database. These analyses consistently demonstrated significant differential expression of CSPG4 in BLCA compared to normal tissues, reinforcing the statistical significance of this observation (Figure 6A, 6B). Furthermore, we investigated the correlation between CSPG4 expression and OS as well as progression-free survival among patients with BLCA. The results revealed that patients with low CSPG4 expression exhibited significantly improved OS and progression-free survival rates compared to those with high CSPG4 expression. This suggests that elevated CSPG4 expression within BLCA is indicative of a poorer prognosis (Figure 6C, 6D). In the stacked bar chart in Figure 6E, a clear trend emerged, with a higher number of deaths and a lower number of survivors (n = 203) observed among patients with BLCA exhibiting high CSPG4 expression. Additionally, we constructed nomogram and OS nomogram models based on the results of a multivariate Cox regression analysis of CSPG4 expression. These models served to elucidate the impact of CSPG4 on the prognosis of patients with BLCA more comprehensively (Figure 6F, 6G). Finally, we conducted a differential analysis of CSPG4 protein expression between BLCA and normal tissues using the Human Protein Atlas website. This analysis revealed a significantly higher staining intensity of CSPG4 protein in normal tissues compared to BLCA tissues (Figure 6H).

### Expression of CSPG4 and its prognostic significance in BLCA

**Figure 7:**
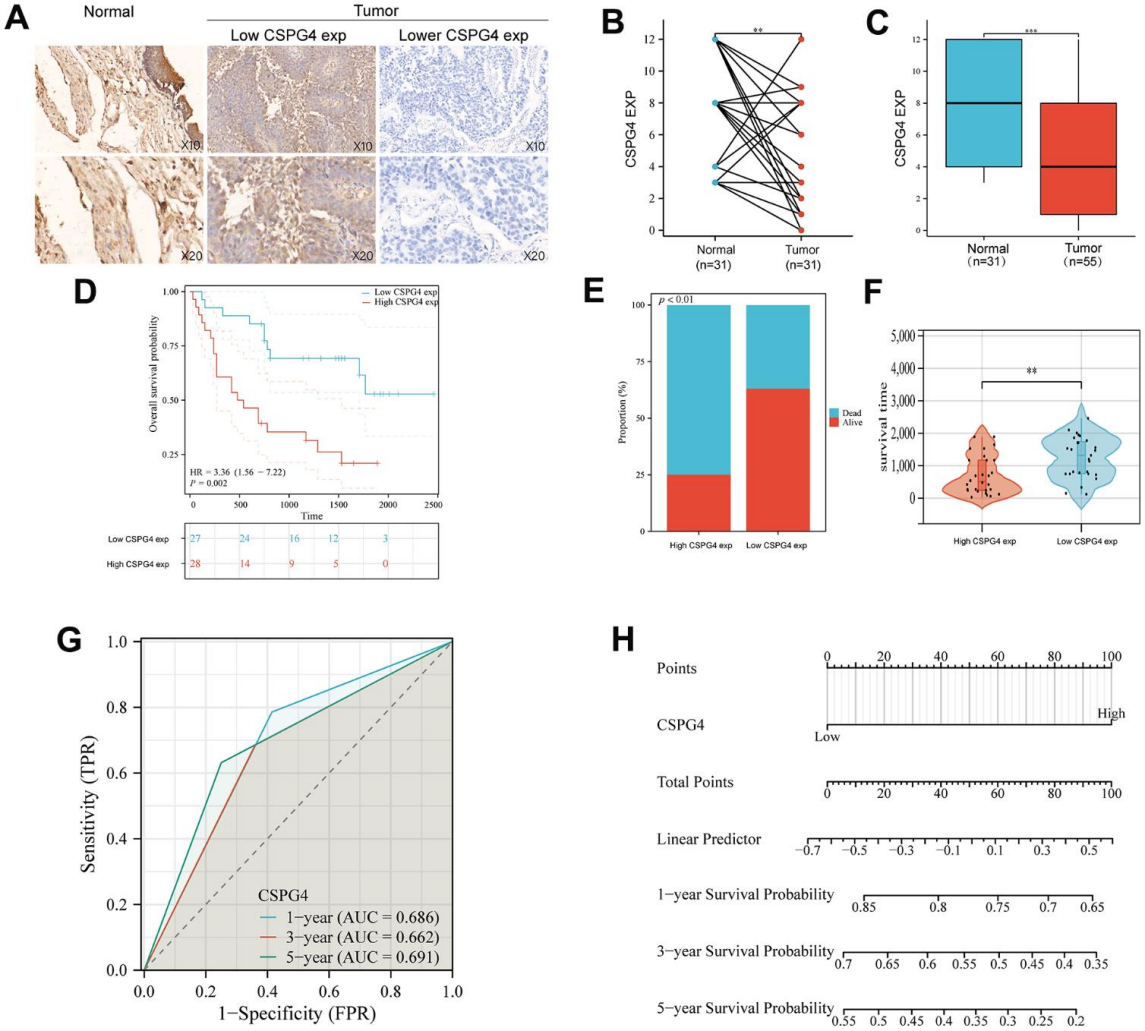
**Expression of CSPG4 in BLCA and its prognostic validation in patients with BLCA.** (**A**) Immunohistochemical validation of CSPG4 expression in bladder tumors and adjacent normal tissues. (**B**) CSPG4 expression in bladder tumors and paired normal tissues. (**C**) CSPG4 expression in bladder tumors and unpaired normal tissues. (**D**) Prognostic analysis of CSPG4 expression in patients with BLCA. (**E**) Analysis of the survival and death rate in patients with BLCA exhibiting high and low CSPG4 expression. (**F**) Impact of CSPG4 expression on survival in patients with BLCA. (**G**) Predictive analysis of the survival of patients with BLCA in the next one, three, and five years based on CSPG4 expression. (**H**) Nomogram based on CSPG4 expression for the prognosis of patients with BLCA in the next one, three, and five years.

To validate our analysis results, we utilized microchip data pertaining to 55 cases of bladder cancer and 31 cases of normal bladder tissue, along with corresponding patient prognostic data. Data pertaining to immunohistochemistry of tissue microarrays revealed that CSPG4 exhibited significantly deeper staining in normal bladder tissue compared to tumor tissue, consistent with our prior analysis (Figure 7A). Furthermore, CSPG4 expression was assessed in 31 BLCA tissues along with paired normal tissues, validating higher CSPG4 expression in normal bladder tissue, corroborating our findings in Figure 5 (Figure 7B). In unpaired BLCA tissue and normal bladder tissue, CSPG4 expression was similarly significantly higher in normal bladder tissue (Figure 7C). In addition, we generated survival curves based on CSPG4 expression and patient outcomes. The graphical representation clearly demonstrated a significantly improved prognosis for patients with low CSPG4 expression (Figure 7D). We additionally constructed a stacked bar chart, which indicated a notably higher ratio of deceased to surviving patients in the high CSPG4 expression group compared to the low expression group (Figure 7E). A violin plot highlighted significantly extended survival times for patients with low CSPG4 expression (Figure 7F). Moreover, CSPG4 exhibited robust predictive capability, enabling us to forecast patient survival at one-, three-, and five-year intervals (Figure 7G). Utilizing a nomogram, we predicted the prognostic impact of high CSPG4 expression on patients with BLCA over the next one, three, and five years, revealing a poorer prognosis for those with high CSPG4 expression (Figure 7H). Finally, we examined the correlation between CSPG4 and clinicopathological factors in patients with BLCA and identified an association between CSPG4 and tumor stage. Univariate and multivariate Cox regression analyses were conducted to investigate the influence of CSPG4 and pathological parameters on the prognosis of patients with BLCA. Univariate analysis demonstrated a correlation between patient prognosis and tumor stage (p = 0.023) as well as CSPG4 expression (p = 0.002). However, multifactorial analysis indicated that only CSPG4 expression (p = 0.024) remained significantly associated with patient prognosis (Tables 1, 2). Therefore, CSPG4 emerges as an independent prognostic factor for patients with BLCA.

**Table 1:** Correlation between CSPG4 and clinicopathological factors of patients with BLCA.

**Characteristics**	**High CSPG4 expression**	**Low CSPG4 expression**	**P-value**
n	28	27	
Age, n (%)			0.925
>65	19 (34.5%)	18 (32.7%)	
≤65	9 (16.4%)	9 (16.4%)	
Gender, n (%)			1.000
male	23 (41.8%)	23 (41.8%)	
female	5 (9.1%)	4 (7.3%)	
Tumor size, n (%)			0.350
>3cm	10 (18.2%)	13 (23.6%)	
≤3cm	18 (32.7%)	14 (25.5%)	
Tumor stage, n (%)			**0.022**
>T2	18 (32.7%)	9 (16.4%)	
≤T2	10 (18.2%)	18 (32.7%)	
Tumor grade, n (%)			1.000
high	24 (43.6%)	24 (43.6%)	
low	4 (7.3%)	3 (5.5%)	
Vascular invasion, n (%)			0.226
Yes	9 (16.4%)	13 (23.6%)	
No	19 (34.5%)	14 (25.5%)	
Lymph node metastasis, n (%)			0.561
Yes	7 (12.7%)	5 (9.1%)	
No	21 (38.2%)	22 (40%)	

**Table 2:** Univariate and multifactorial Cox regression analyses of the effect of CSPG4 and pathological parameters on the prognosis in patients with BLCA.

**Characteristics**	**Total (N)**	**Univariate analysis**			**Multivariate analysis**	
		**Hazard ratio (95% CI)**	**P-value**		**Hazard ratio (95% CI)**	**P-value**
Age	55		0.751			
>65	37	Reference				
≤65	18	1.128 (0.539 - 2.358)	0.749			
Gender	55		0.512			
male	46	Reference				
female	9	1.362 (0.556 - 3.333)	0.499			
Tumor size	55		0.151			
>3cm	23	Reference				
≤3cm	32	1.715 (0.809 - 3.634)	0.160			
Tumor stage	55		**0.021**			
>T2	27	Reference			Reference	
≤T2	28	0.430 (0.207 - 0.890)	**0.023**		0.587 (0.262 - 1.314)	0.195
Tumor grade	55		0.562			
high	48	Reference				
low	7	0.713 (0.216 - 2.356)	0.579			
Vascular invasion	55		0.164			
Yes	22	Reference				
No	33	1.692 (0.792 - 3.617)	0.175			
Lymph node metastasis	55		0.091			
Yes	12	Reference			Reference	
No	43	0.489 (0.223 - 1.075)	0.075		0.469 (0.210 - 1.045)	0.064
CSPG4	55		**0.001**			
Yes	28	Reference			Reference	
No	27	0.298 (0.139 - 0.639)	**0.002**		0.384 (0.167 - 0.882)	**0.024**

### Correlation between CSPG4 and the immune microenvironment in BLCA

**Figure 8:**
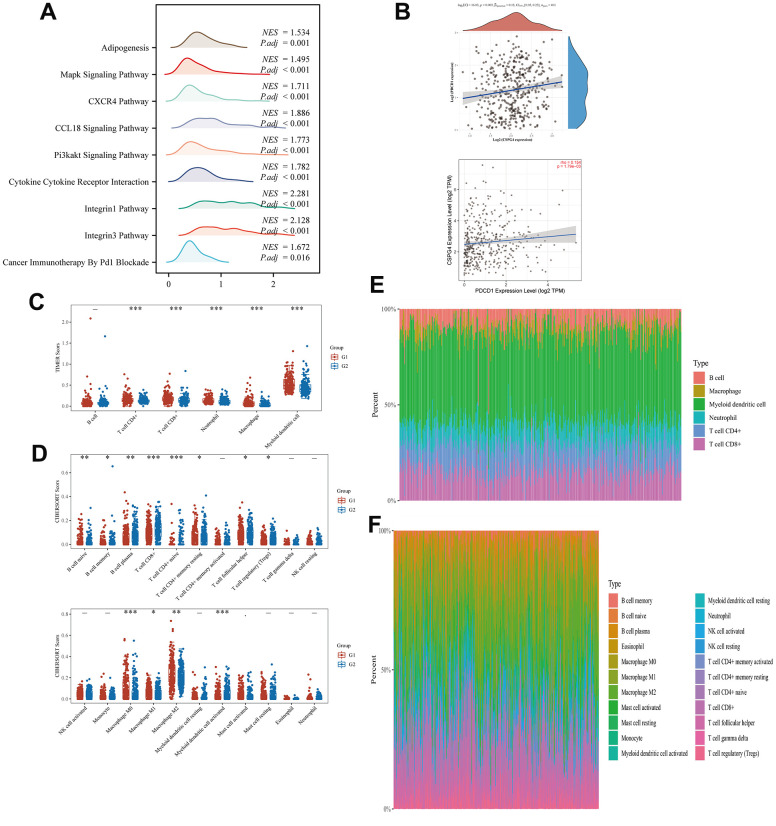
**Correlation between CSPG4 and the BLCA immune microenvironment.** (**A**) Ridge plot showing the association between CSPG4 and the BLCA immune microenvironment. (**B**) Correlation of CSPG4 with PD1 expression. (**C**, **D**) Differential expression of immune cells between high and low CSPG4 expression groups. (**E**, **F**) Heatmap of the percentage of immune infiltrating cells in the high and low CSPG4 expression groups.

We performed GSEA to assess the enrichment patterns of CSPG4 in high- and low-expression groups of BLCA. The results of our analysis, visualized as a ridge plot, revealed a significant association between CSPG4 and “cancer immunotherapy by PD1 blockade” in the BLCA immune microenvironment. This underscores the significance of PD1 as a crucial target for BLCA immunotherapy owing to the interrelationship between CSPG4 and PD1 (Figure 8A). Subsequently, we examined the correlation between CSPG4 and PD1, finding a positive association in their expression levels (Figure 8B). Additionally, we explored variations in immune cell expression between the high and low CSPG4 expression groups using the TIMER and CIBERSORT algorithms. The results indicated that T cells, B cells, Natural Killer (NK) cells, macrophages, and monocytes were expressed in both groups, although without significant differences in expression levels (Figure 8C, 8D). Furthermore, we generated a heatmap illustrating the percentages of immune infiltrating cells in both groups, validating a positive correlation between CSPG4 and PD1 expression (Figure 8E, 8F). In summary, CSPG4 plays a pivotal role in the immune microenvironment of BLCA and holds substantial relevance to PD1 immunotherapy in patients with BLCA.

## DISCUSSION

BLCA, ranking as the tenth most prevalent tumor globally, has garnered significant attention in the context of treatment strategies. Immunotherapy stands out as a crucial approach for the management of muscle-invasive bladder cancer, with its efficacy well established. Notably, immunotherapy's effectiveness is intricately linked to the TIME. Therefore, a comprehensive exploration of TIME holds significant guidance potential for advancing BLCA immunotherapy [34, 35]. ICI therapies, such as PD1 inhibition and PD-L1 inhibition, are prominent in BLCA treatment, particularly for patients in advanced stages [36]. Aberrations in glycolysis and, consequently, energy metabolism induced by EMT have been found to correlate with TIME irregularities, thereby influencing the resistance to immunotherapy in tumors [37]. However, the role played by EMT and energy metabolism within the BLCA tumor microenvironment remains incompletely understood.

In this study, we conducted a comprehensive investigation utilizing TCGA database, focusing on CSPG4, a pivotal gene associated with EMT and energy metabolism. We observed differential expression of CSPG4 between BLCA tissues and normal tissues, establishing its significant associations with prognosis in patients with BLCA, the BLCA immune microenvironment, and PD1 immunotherapy outcomes for patients with BLCA. A recent investigation elucidated CSPG4's role as a polymeric membrane proteoglycan, promoting tumor proliferation and metastasis through the tyrosine kinase pathway while also influencing tumor energy metabolism to facilitate neovascularization [38]. In addition, CSPG4 affects EMT-related pathways by influencing extracellular matrix receptor interactions, extracellular matrix disassembly, and extracellular matrix assembly, thereby affecting tumor progression. Notably, the knockdown of CSPG4 significantly inhibits the expression of PD-L1 [39]. However, the pivotal role of CSPG4 in BLCA, particularly concerning EMT and energy metabolism, remains incompletely explored. Here, we conducted a comprehensive analysis to elucidate the significant association between CSPG4 and BLCA prognosis, as well as its influence on immune infiltration. EMT is initiated by various stimuli, such as cytoskeletal rearrangements and extracellular matrix remodeling, enabling cells to acquire invasive mesenchymal characteristics [40]. Moreover, EMT has been linked to the induction of tumor cell autophagy through Ras, Wnt, and NF-κβ signaling pathways, consequently promoting tumor migration by modulating tumor cell energy metabolism [41]. Alterations in glycolysis, oxidative phosphorylation, and lipid metabolic pathways in tumor cells can result in abnormal energy metabolism, subsequently regulating cellular EMT and contributing to tumor progression. Furthermore, PD1 expression has been observed to exhibit a correlation with both EMT and energy metabolism within the TIME [42]. Therefore, we posit that EMT and energy metabolism play pivotal roles in tumor progression and are intricately linked to the TIME, thereby exerting a profound influence on the efficacy of tumor immunotherapy. Research has demonstrated a close association between EMT, energy metabolism, and BLCA. Dysregulated cellular energy metabolism, mediated through the Phosphatidylinositol 3 Kinase/ Automatischer Kassentresor (PI3K/AKT) signaling pathway, induces EMT in BLCA cells, consequently promoting their proliferation and invasion [43]. Additionally, within the BLCA immune microenvironment, it has been observed that exosomes have the capacity to disrupt energy metabolism through their cargo of glucose, proteins, and lipids, thereby facilitating the EMT process [44]. Therefore, we posit that both EMT and energy metabolism hold significant relevance within the context of the BLCA immune microenvironment. In addition, EMT- and energy metabolism-related genes have been demonstrated to exert a substantial impact on the prognosis of patients with BLCA, thereby emerging as potential prognostic biomarkers for BLCA [45, 46]. Inhibiting EMT, including the suppression of mesenchymal marker N-calmodulin and EMT-related pathways such as TGF-β and Wnt/β-catenin, has been shown to significantly impede the EMT process, leading to substantial inhibition of BLCA invasion and metastasis [47]. Similarly, by targeting tumor cell glycolysis to regulate energy metabolism, a significant reduction in the invasiveness of BLCA has been observed [48]. Thus, it is evident that both EMT and energy metabolism play pivotal roles in BLCA.

In the context of BLCA, the selection of appropriate EMT- and energy metabolism-related targets assumes paramount significance for optimizing late-stage treatments and enhancing the prognosis of patients with BLCA [49, 50]. In this study, we stratified patients with BLCA into two distinct groups based on the expression profiles of genes associated with EMT and energy metabolism, employing a consensus cluster analysis. Our findings revealed significant disparities between these two patient cohorts, with Group 2 exhibiting significantly improved BLCA prognoses in comparison to Group 1. This observation substantiates the differential expression of EMT- and energy metabolism-related genes in BLCA. To identify the most significant genes associated with EMT and energy metabolism in BLCA, we first screened 22 genes that have relevance to both EMT and energy metabolism, utilizing data from TCGA. Subsequently, we performed gene intersection analysis to pinpoint six genes, namely *HS3ST3B1*, *FMOD*, *CSPG4*, *ALDH1A1*, *CYP1B1*, and *ENO1*, which demonstrated associations with BLCA prognosis and immune infiltration among the pool of EMT- and energy metabolism-related genes.

CSPG4 emerged as a pivotal gene in the context of EMT and energy metabolism in BLCA. Our comprehensive investigation, encompassing analyses of immunoinfiltration, prognosis, pathological staging, grade, and paired samples, underscored its potential as a promising prognostic biomarker for patients with BLCA. Furthermore, we explored the disparities in CSPG4 expression between BLCA and normal tissues, alongside an assessment of CSPG4's prognostic significance in patients with BLCA, substantiated through immunohistochemistry and clinical patient data. Heightened CSPG4 expression is correlated with an unfavorable prognosis in patients with BLCA. Moreover, through GSEA, we found that PD1 blockade cancer immunotherapy directly enriched CSPG4. This finding implies a substantial correlation between CSPG4 and PD1. However, the precise mechanisms underlying CSPG4's involvement in the immune microenvironment remain incompletely elucidated. Nevertheless, this study establishes a research foundation and theoretical framework for further investigations into the potential role of CSPG4 in BLCA immunotherapy.

## CONCLUSIONS

In this study, we comprehensively analyzed EMT- and energy metabolism-related genes in BLCA. We divided BLCA samples from TCGA into two groups via clustering analysis and then assessed the expression differences of relevant genes between the distinct tumor samples and investigated their prognostic implications. We identified CSPG4 as the most pivotal gene, which was subsequently validated to be differentially expressed in BLCA through analytical methods such as Cox regression analysis. Patients with BLCA exhibiting high CSPG4 expression had a poorer prognosis. Furthermore, we discovered a correlation between CSPG4 expression and PD1 expression within the immune microenvironment of BLCA. However, the precise underlying mechanism driving the relationship between CSPG4 and PD1 expression, as well as the prognosis associated with PD1 immunotherapy in patients with BLCA, warrants further investigation through fundamental experimental and clinical trial analyses.
